# Zebrafish lipid droplets regulate embryonic ATP homeostasis to power early development

**DOI:** 10.1098/rsob.170063

**Published:** 2017-07-05

**Authors:** Asmita Dutta, Deepak Kumar Sinha

**Affiliations:** Department of Biological Chemistry, Indian Association for the Cultivation of Science, Jadavpur, Kolkata 700032, India

**Keywords:** zebrafish embryos, lipid droplets, lipolysis, free fatty acids, embryonic ATP, active protein degradation

## Abstract

In zebrafish embryos, the maternally supplied pool of ATP is insufficient to power even the earliest of developmental events (0–3 hpf) such as oocyte-to-embryo transition (OET). The embryos generate an additional pulse (2.5 h long) of ATP (1.25–4 hpf) to achieve the embryonic ATP homeostasis. We demonstrate that the additional pulse of ATP is needed for successful execution of OET. The maternally supplied yolk lipids play a crucial role in maintaining the embryonic ATP homeostasis. In this paper, we identify the source and trafficking routes of free fatty acids (FFAs) that feed the mitochondria for synthesis of ATP. Interestingly, neither the maternally supplied pool of yolk-FFA nor the yolk-FACoA (fatty acyl coenzyme A) is used for ATP homeostasis during 0–5 hpf in zebrafish embryos. With the help of lipidomics, we explore the link between lipid droplet (LD)-mediated lipolysis and ATP homeostasis in zebrafish embryos. Until 5 hpf, the embryonic LDs undergo extensive lipolysis that generates FFAs. We demonstrate that these newly synthesized FFAs from LDs are involved in the maintenance of embryonic ATP homeostasis, rather than the FFAs/FACoA present in the yolk. Thus, the LDs are vital embryonic organelles that maintain the ATP homeostasis during early developmental stages (0–5 hpf) in zebrafish embryos. Our study highlights the important roles carried on by the LDs during the early development of the zebrafish embryos.

## 1. Introduction

**Figure 1.**
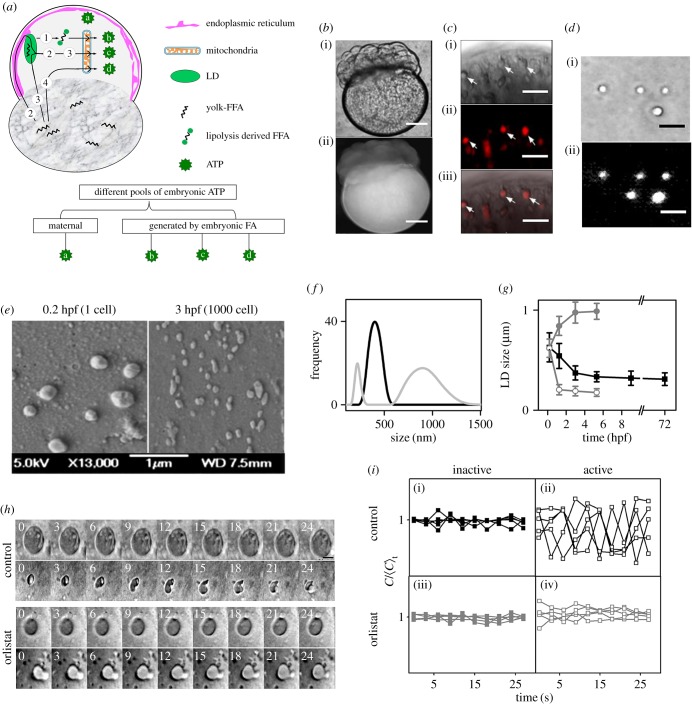
Blastomeric LDs in the zebrafish embryos undergo extensive lipolysis during early development (0–3 hpf). (*a*) Schematic diagram showing probable routes of ATP generation by mitochondrial β-oxidation of precursor FFAs generated from either the yolk or the blastodisc or LDs during zebrafish embryonic development. (*b*) (i) DIC and (ii) fluorescence images of 16–32 cell stage embryo fixed and stained with NR showing the blastodisc and the yolk regions. (*c*) (i) DIC, (ii) fluorescence and (iii) merged images of embryo blastodisc showing LDs marked with arrows. (*d*) (i) DIC and (ii) fluorescence images of LDs isolated from blastodiscs of zebrafish embryos and stained with NR. (*e*) SEM images of LDs isolated from the blastodiscs of embryos at 0.2 hpf (left panel) and 3 hpf (right panel). (*f*) Size distribution plots of LDs isolated from control embryos (black) and OS-treated embryos (grey) both at 1000-cell stage. (*g*) Average size of LDs obtained from embryos at different developmental stages plotted across respective time points for control (black) and OS-treated embryos (grey). Open and closed symbols signify two distinct populations of LDs isolated from OS-treated embryos. Error bars are standard error of means (s.e.m.). (*h*) Time-lapse images of representative LDs in control (upper two panels) and OS-treated (lower two panels) embryos. Upper panel stamps of both control and OS-treated embryos denote inactive state, while the lower panel stamps denote the active state under each condition. Time stamps are in seconds. (*i*) Upper panel denotes five representative time traces of normalized circularity values of LDs in control embryos (black) during (i) inactive state and (ii) active state. Lower panel denotes time traces of normalized circularity values of LDs in OS-treated embryos (grey) during (iii) inactive and (iv) active states. Scale bars: 100 µm in (*b*), 1 µm in (*c*,*d*) and 0.25 µm in (*h*).

The precision of embryonic development into an adult depends on the underlying biochemical reaction network. The meticulous execution of such a complex spatio-temporal reaction network is powered primarily by the ATP molecules. Therefore, maintaining the homeostasis of ATP is vital for proper embryonic development. The mammalian pre-implantation embryos use ions and nutrients such as pyruvate, glucose, lactate and amino acids in the oviduct to achieve ATP homeostasis [1]. However, the lecithotrophic (feeding on yolk) embryos depend on the yolk lipids for ATP homeostasis until they start feeding from external sources [2]. Various literature reports have established that oxidation of yolk free fatty acids (FFAs) in pythons [3], reptiles [2] and birds [4–6] fulfil the ATP needs of these lecithotrophic embryos. However, these studies are performed at later developmental stages such as hatching (20–59 days post-fertilization for different organisms). During the very early stages of development, such as first few hours of fertilization, the embryos contain a maternally supplied pool of ATP. Thus, the oxidation of FFA may not be as relevant during early stages of development as it is during later stages for ATP homeostasis. The duration for which the maternal pool of ATP (pool a, figure 1*a*) can sustain the zebrafish embryonic development is not known. Whether ATP derived from β-oxidation of embryonic FFAs is needed right from the time of fertilization or is it required at later stages of development needs to be investigated. Oocyte-to-embryo transition (OET) is one of the earliest developmental events that involves ATP-dependent degradation of maternal proteins in the embryos [7]. Thus, studying the dependence of OET on maternally stored pool of ATP could answer such questions.

In addition to FFAs, the yolk also contains other forms of neutral lipids (NLs) such as triacylglycerols (TAGs), cholesterol esters (CEs), diacylglycerol (DAG) and wax [8,9], whose lipolysis could generate FFA to power the ATP homeostasis in lecithotrophic embryos. The role of other NLs in the yolk with regard to ATP homeostasis in lecithotrophic embryos is not known. Lipid droplets (LDs), reservoirs for hydrophobic molecules within the cytoplasm, are studded with lipases such as adipose triglyceride lipase (ATGL), hormone-sensitive lipase (HSL) [10–13]. This makes LDs an NL processing factory in the cell which can generate FFAs from breakdown of NLs [10]. The FFA obtained via lipolysis of LDs could serve as the fuel for ATP synthesis via β-oxidation pathway [14,15]. Because of various non-canonical functions of LDs observed in cells [16–20], we believe that their involvement in embryogenesis could be beyond the storage of hydrophobic molecules. Though the association of LDs with many human diseases [21,22] has propelled them into the limelight as important cellular organelles, yet their role in the normal physiology of cells and the development of an embryo needs more attention.

In lecithotrophic zebrafish embryos [23], the NLs present in the yolk serve as fuel for metabolic energy production [9]. We propose two distinct sources of FFAs that might fulfil the needs of embryonic ATP synthesis: (i) the maternally stored FFAs in the yolk and (ii) the FFA generated by breakdown of other NLs in LDs via lipolysis (figure 1*a*, route 1). Either the yolk-FFAs migrate to the mitochondria via LDs [23] (figure 1*a*, route 2 and route 3) or they migrate directly to the mitochondria (figure 1*a*, route 4). Whether the yolk-FFAs present within the LDs (figure 1*a*, route 2 and route 3) are used to generate embryonic ATP or the new FFAs generated by lipolysis of the LD (figure 1*a*, route 1) are fed to mitochondria is not known. In this paper, we identify the pool of FFAs that is used for the synthesis of embryonic ATP during early embryogenesis. Thus, based on its source, we expect two distinct pools of embryonic ATP (i) maternally stored (pool a, figure 1*a*) and (ii) synthesized from β-oxidation of embryonic FFAs (pool b, pool c and pool d, figure 1*a*). The second pool of ATP can be further categorized into two distinct classes: (i) derived from FFAs generated from lipolysis of NLs of the LDs (pool b, figure 1*a*, route 1) and (ii) derived from yolk-FFAs (pool-c and pool-d via: routes 2, 3 and 4).

Zebrafish embryos contain at least 15 different types of lipase transcripts [25,26] (table 1). As the surfaces of the LDs are embedded with a large number of lipases and lipid synthases, one requires simultaneous genetic knockdown of a class of lipases to elucidate the role of LD metabolism in the development of embryos. Additionally, morpholino-based proteomic manipulation takes 1–3 days to show a desirable phenotype in zebrafish embryos [27,28]. Hence, such manipulations cannot be used for functional knockdown of a class of gene family during early stages of embryonic development. Therefore, the pharmacological approach of functionally controlling a class of genes is the most appropriate method to elucidate the role of LD metabolism during early development of the zebrafish embryos.

**Table 1.** Classes of lipase enzymes present in zebrafish embryos at different developmental stages.

lipase	gene in zf	2 hpf	64 hpf	3.5 hpf	6 hpf	9 hpf
carboxyl ester lipase, CEL	Cel.2	0	0	0.3067	0.3256	0.2992
hormone-sensitive lipase, HSL	LIPE.A	0.1769	1.3927	0.8844	0	0.0913
	LIPE.B	0.6616	0.6254	0.5482	0.2654	0.5042
gastric lipase, LIPF	LIPF	1.8493	4.0303	2.7932	0.4932	0.9589
endothelial lipase	LIPG	150.75	14.6434	9.6828	0.5932	0.3739
lipoprotein lipase	LPL	0	0.0868	0.4274	4.2457	1.0876
monoglyceride lipase	MGLL	0.0398	0.0957	8.2463	81.069	108.0285
diacylglycerol lipase	AADACL-4	0.14	0.17	0.29	8.7	41.87
patatin-like phospholipase domain containing 2	PNPLA7a	36.876	29.333	28.984	12.347	2.75
	PNPLA7b	1.4407	1.9040	1.9009	3.9695	15.2603
	PNPLA4	0.2339	1.7893	1.2908	0.4944	1.5506
	PNPLA2	0.8426	1.4783	1.1749	0	0
	PNPLA8	2.8005	11.8518	4.8719	0	0.1661
	PNPLA1	0	0	0.0603	0.3015	0.3283
	PNPLA6	0	0	0.0604	0.3014	0.3283

In this paper, we demonstrate that the maternally supplied pool of ATP in the embryo is insufficient to power the very early stages of development. Thus, we investigate the proposed routes of yolk-FFAs trafficking by injection of Bodipy-labelled oleic acid. The proposed pools of ATP have also been explored by pharmacological perturbation of lipolysis. We establish that in spite of the presence of sufficient FFAs stored in the yolk, in the early stages of embryogenesis, the FFAs involved in maintaining the ATP homeostasis are derived by lipolysis of the LDs. This highlights an important function of LDs in zebrafish early development.

## 2. Results

### 2.1. LDs are localized exclusively in the blastodisc of the zebrafish embryos

We examined the fluorescence image of live zebrafish embryos stained with lipophilic dye, Nile red (NR) [29]. In agreement with previous reports that yolk contains NLs and FFAs, we observe a stronger fluorescence of NR in the yolk than in the blastodisc (figure 1*b*). To visualize the presence of LDs, we further examined the blastodisc (figure 1*c*) and the yolk (data not shown) under higher optical magnification. We observe punctate structures within the blastodisc which stain with NR (figure 1*c*). This suggests that the NLs/FFAs within the blastodisc region are stored in the form of LDs. However, even under higher magnification, we do not observe any fluorescently stained distinct punctate structures within the yolk. Therefore, it is not clear whether the NLs/FFAs present within the yolk are stored in the form of LDs. To further test the presence of LDs in the yolk, we separated the yolk from the blastodisc. We analysed both the fractions (blastodisc and yolk) for the presence of LDs using previously reported biochemical LD isolation techniques [30,31] with few modifications. Figure 1*d* shows the image of LDs isolated from blastodisc fraction which gets strongly stained with NR. Though the yolk fraction contains a lot of lipids, yet we failed to observe any distinct granular structure in the appropriate sucrose layer (electronic supplementary material, figure S1). This shows that the LDs exist exclusively in the blastomere region of the zebrafish embryos.

### 2.2. Lipolysis reduces the size of LDs during early development of zebrafish embryos

We compared the size of LDs isolated from embryos at 0.2 hpf (1-cell stage) and 3 hpf (1000-cell stage) using scanning electron microscopy (SEM). We observe a marked decrease in the LDs' size at 3 hpf (average size ∼ 320 nm) compared with LDs from 0.2 hpf embryos (average size ∼ 600 nm; figure 1*e*). As SEM imaging involves chemical fixing of the LDs using aldehydes, which could lead to alterations in the LDs' size, we confirmed the LDs' size by dynamic light scattering (DLS) measurement (which does not involve any fixing). The DLS measurement yielded similar size of LDs as obtained from the SEM images (average size from DLS at 1000-cell stage is 373.03 ± 70.42 nm; figure 1*f*). Having established that DLS and SEM results are similar, we explored the LDs' size at multiple developmental stages using DLS (figure 1*g*). We found that the size of the LDs decreases steadily up to 3 hpf. To confirm that lipolysis of LDs leads to decrease in LD size, we treated the embryos with a lipase inhibitor, Orlistat (OS), which is known to inhibit the activities of gastric and intestinal lipases [32,33]. Inhibition of lipolysis generates two differently sized populations of LDs, unlike control embryos which had a single-sized population (figure 1*f,g*). This suggests that the size of the LDs during early development of zebrafish embryos is reduced mainly by the active metabolism of the LDs.

### 2.3. Lipolysis drives shape fluctuation of LDs in zebrafish embryos

As reported by us previously, the LDs present in the blastodisc of zebrafish embryos exhibit two distinct states, namely the ‘inactive’ state when the LDs are bigger, circular and stable, while in the ‘active’ state, the LDs are smaller, irregularly shaped and structurally unstable [34]. We compared the fluctuation of the LDs' shape over time. 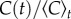 defines the time averaged circularity of the LDs (see ‘Experimental procedures’) in control compared with that of the OS-treated embryos (figure 1*h,i*). We observe (figure 1*h,i*(ii)(iv)) that inhibition of lipolysis by OS reduces the shape fluctuation of LDs during active state. On the other hand, inhibition of lipolysis has no measurable effect on the shape fluctuation during the ‘inactive’ state (figure 1*h,i*(i)(iii)), suggesting that the biochemical activity of lipases generates enough mechanical stress within the LDs that deform their shape.

### 2.4. Lipolysis alters the lipid composition of the LDs during early embryonic development

**Figure 2.**
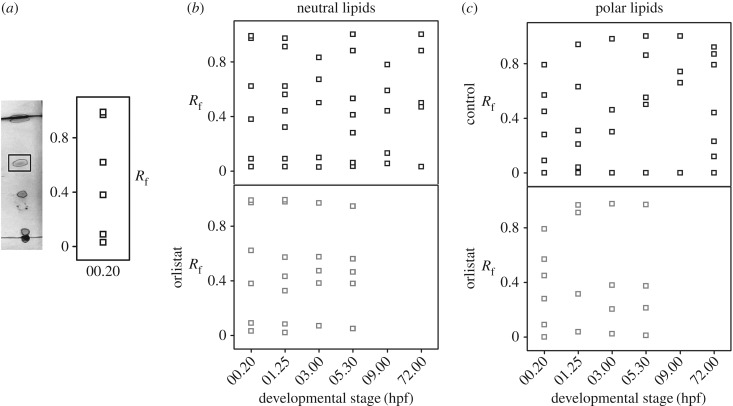
LD metabolism alters its lipid profile. (*a*) Left panel: TLC profile of NLs extracted from LDs of normal embryos at 20 mpf (minutes post-fertilization). Right panel: plot of corresponding *R*_f_ values for each TLC spot plotted against developmental time. (*b*) Plot of *R*_f_ of lipids extracted from LDs of control embryos (upper panel) and OS-treated embryos (lower panel) when run in neutral solvent system versus corresponding developmental stages of the embryos. (*c*) Plot of *R*_f_ of lipids extracted from LDs of control embryos (upper panel) and OS-treated embryos (lower panel) when run in polar solvent system versus corresponding developmental stages of the embryos. All TLC experiments were done in triplicates and only reproducible TLC patterns have been reported.

We next investigated if lipolysis also alters the lipid composition of the LDs. Using thin layer chromatography (TLC), we compared the lipid profile of the LDs isolated from embryos at different developmental stages. As lipids differ in their polarities, they migrate to different distances on the TLC plates depending upon the solvent system used for the specific TLC run (see ‘Experimental procedures’). We used two different solvent systems (neutral and polar solvent) to resolve the neutral (TAGs, CEs, etc.) and the polar lipids (like PLs, cholesterols, etc.) extracted from the LDs. We extracted lipids from the LDs isolated from the embryos at different stages of development. Figure 2*a* (left panel) shows a representative TLC profile of the LD-extracted lipids (LDls) from 1-cell-stage embryos (00.20 hpf) run in neutral solvent. As shown in figure 2*a* (right panel), we calculated the resolving fraction (*R*_f_) for each spot from this TLC plate. *R*_f_ denotes the migration of the particular compound to the migration of the solvent front. Each spot on the TLC plate with unique *R*_f_ value corresponds to different lipid classes (see ‘Experimental procedures’). Moreover, each spot on the TLC plate may contain multiple lipids with similar polarity. To resolve this further, one needs to use mass spectrometry-based analysis for each spot. We observe a reproducible unique pattern of LDls at each developmental stage, suggesting that the NL and polar lipid contents of the LDs vary as the embryo develops from 0.2 hpf (1-cell stage) to 3 dpf (larvae) (figure 2*b,c*; electronic supplementary material, figure S2). Thus, the lipid composition of LDs gradually changes during embryonic development. Next, we explored if the observed change in the profile of the LDls is driven by lipolysis. For this, we studied the LDls in OS-treated embryos. In these embryos, the lipid profiles for both neutral and polar lipids do alter up to 3 hpf (figure 2*b,c*). However, beyond this stage, we do not observe any significant changes in the LDls (figure 2*b,c*), suggesting that metabolism of the LDs alters the LDl composition during early embryonic development. Table 2 gives the list of different lipid types and their corresponding *R*_f_ values when TLC is run in neutral [35] and polar [36] solvent systems. Table 3 summarizes the LDl composition (as identified by TLC) at different developmental stages in control and OS-treated embryos. The name of lipids provided in table 3 is assigned using the *R*_f_ value information provided in table 2.

**Table 2.** List of lipid classes and their corresponding TLC-*R*_f_ values as assembled from literature reports. *R*_f_ values of lipids when TLC is run in neutral [32] and polar solvents [33]. MAG, monoacylglycerols; DAG, diacylglycerols; TAG, triacylglycerols; FFA, free fatty acids; STE, sterol esters; SQD, sulfoquinovosyldiacylglycerol; DGD, digalactosyldiacylglycerol; CER, cerebrosides; SG, steryl glucoside; MGD, monogalactosyldiaclyglycerol; ESG, esterified steryl glucoside; PS, phophatidylserine; PI, phosphatidylinositol; PC, phosphatidylcholine; PE, phosphatidylethanolamine; ST, sterol lipids.

neutral solvent		polar solvent	
*R*_f_	neutral lipids	*R*_f_	glycolipids
0.09–0.14	MAG	0.06	SQD
0.3–0.32	DAG	0.17	DGD
0.37–0.45	ST	0.28–0.35	CER
0.54–0.64	FFA	0.41	SG
0.77–0.88	TAG	0.62–0.66	MGD
0.95	STE	0.76	ESG

		*R*_f_	phospholipid
		0.04	PS
		0.11–0.13	PI
		0.20–0.23	PC
		0.30–0.31	PE

**Table 3.** LD-associated lipids in control and OS-treated embryos at different developmental stages. *R*_f_ for each lipid spot has been calculated and then tallied with data from table 2 to determine the lipid class to which each of the lipid types they belong to. MAG, monoacylglycerols; DAG, diacylglycrols; TAG, triacylglycerols; FFA, free fatty acids; STE, sterol esters; CER, cerebrosides; MGD, monogalactosyldiaclyglycerol; PS, phophatidylserine; PI, phosphatidylinositol; PC, phosphatidylcholine; PE, phosphatidylethanolamine; ST, sterol lipids; ‘—’ denotes the *R*_f_ values for which no lipid classes have been identified in this solvent.

	neutral solvent				polar solvent			
	control		OS		control		OS	
developmental stage	*R*_f_	class	*R*_f_	class	*R*_f_	class	*R*_f_	class
1 cell	0.09	MAG	0.09	MAG	0.09	—	0.09	—
	0.38	ST	0.38	ST	0.28	CER	0.28	CER
	0.62	FFA	0.62	FFA	0.45	—	0.45	—
	0.97	STE	0.97	STE	0.57	—	0.57	—
	0.989	STE	0.989	STE	0.79	—	0.79	—
8 cell	0.09	MAG	0.083	—	0.04	PS	0.038	—
	0.32	DAG	0.33	DAG	0.21	PC	0.32	CER
	0.44	ST	0.43	ST	0.31	PE	0.91	—
	0.56	FFA	0.57	FFA	0.63	MGD	0.97	—
	0.62	FFA	0.95	STE	0.94	—		
	0.91	STE	0.99	—				
	0.97	STE						
1000 cell	0.10	MAG	0.07	—	0.3	CER	0.023	—
	0.54	FFA	0.38	ST	0.46	—	0.21	PC
	0.64	FFA	0.47	—	0.98	—	0.38	—
	0.83	TAG	0.57	FFA			0.97	—
			0.97	—				
50% epiboly	0.06	—	0.05	—	0.5	—	0.013	PI
	0.30	DAG	0.38	ST	0.55	—	0.21	PC
	0.41	ST	0.46	—	0.86	—	0.37	—
	0.53	FFA	0.56	FFA	1	—	0.97	—
	0.88	TAG	0.95	STE				
90% epiboly	0.13	MAG			0.66	MGD		
	0.44	ST			0.74	—		
	0.59	FFA			1	—		
	0.78	TAG						
3 dpf	0.032	DAG			0.12	PI		
	0.45	ST			0.23	PC		
	0.50	FFA			0.44	—		
	0.88	TAG			0.79	—		
					0.87	—		
					0.92	—		

### 2.5. LDs present within the blastodisc generate FFAs by lipolysis during early embryogenesis

**Figure 3.**
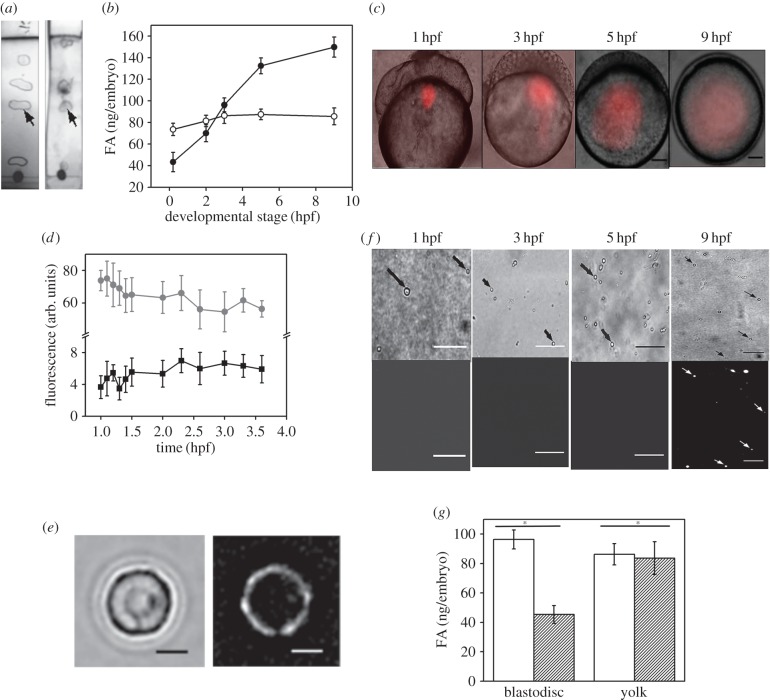
Generation of FFAs in the blastodisc during early zebrafish embryogenesis. (*a*) TLC of lipids extracted from blastodisc (left TLC strip) and yolk (right TLC strip) of 1000-cell stage embryos. Arrows point to the spot corresponding to the *R*_f_ of FFAs (approx. 0.56). (*b*) Plot comparing the FFA content of embryonic blastodisc (closed symbol) and yolk (open symbol) fractions at different stages of embryonic development. Error bar denotes s.e.m. (*c*) Time-lapse DIC images overlaid with the corresponding fluorescence images of embryos injected with Bodipy-C_12_ into the yolk and imaged at different developmental stages over time. Scale bar, 100 µm. (*d*) Plot of fluorescence intensity versus embryo development (in hpf) to compare the changes in the fluorescence signal in the yolk (grey) and blastodisc (black) when Bodipy-C_12_ is injected into the yolk of the embryos at 1-cell stage. Error bar denotes s.e.m. (*e*) Isolated LDs stained *in vitro* with Bodipy-C_12_. Note the ring-like appearance of the LD when stained *in vitro*. Scale bar, 200 nm. (*f*) DIC (top panel) and fluorescence (lower panel) images of LDs isolated from embryos at different developmental stages, injected with Bodipy-C_12_ into the yolk at 1-cell stage. Arrows point to the LDs present in the field of view. Scale bar, 3 µm. (*g*) Plot showing the difference in FFA content of the blastodisc and yolk fractions of control embryos (empty bars) with that of OS-treated embryos (shaded bars) at 1000-cell stage. Result of two-sample *t*-test is given, with asterisk denoting significant difference between the two sample sets and ‘n.s.’ denoting no significant difference between the sample sets. All experiments are done in triplicates and the mean is plotted with s.e.m. as the error bars.

Next, we analysed the presence of FFAs within the yolk and the blastodisc regions of the embryo using TLC. Figure 3*a* shows the composition of the lipids (*R*_f_ values) for the yolk and the blastodisc of embryos at 1000-cell stage. We observe TLC spots (*R*_f_ ∼ 0.56) corresponding to FFA in both the yolk and blastodisc fraction. We further confirmed this by MALDI-TOF mass spectrometry (electronic supplementary material, figure S3 and table S1). As expected, even at the 1-cell stage, the endogenous FFA is present within both the yolk (figure 3*b*; 73.542 ± 5.673 ng embryo^−1^) and the blastodisc (figure 3*b*; 43.289 ± 8.99 ng embryo^−1^) regions of the embryos. To understand the trafficking of endogenous FFAs within the yolk, we injected fluorescently tagged FFA (Bodipy-C_12_) [8] into the yolk. Figure 3*c* depicts the time-lapse images of the embryo injected with exogenous FFA into the yolk near the yolk–blastodisc interface. We expect the total fluorescence of Bodipy-C_12_ within the yolk and the blastodisc to alter as a result of FFA migration. Figure 3*d* depicts no significant migration of yolk-FFA into the blastodisc as estimated by total fluorescence intensity of Bodipy-C_12_. This suggests that there is no trafficking of yolk-FFAs to the blastodisc measurable by fluorescence imaging. However, if a small amount of yolk-FFAs migrate to the blastodisc, we can visualize it by fluorescence labelling of the LDs. The hydrophobic interaction of Bodipy-C_12_ with isolated LDs manifests as surface labelling of the LDs (figure 3*e*). We tested the migration of a small amount of yolk-FFAs by analysing the Bodipy-C_12_ fluorescence of isolated LDs. The exogenous FFAs injected in the yolk do not interact with the LDs until 5 hpf (figure 3*f*), suggesting that the endogenous yolk-FFAs do not migrate to the blastodisc until 5 hpf. Thus, the increase in blastodisc FFAs (figure 3*b*) within the blastodisc during early zebrafish embryogenesis must be fulfilled by lipolysis (i.e. route 1, figure 1*a*). We establish that the generation of new FFAs within the blastodisc is via lipolysis by comparing the levels of FFAs within the blastodisc and the yolk in control embryos with that of the OS-treated embryos. Figure 3*g* establishes that the inhibition of lipolysis lowers the FFA levels within the blastodisc of the zebrafish embryos. As expected, inhibition of lipolysis has no effect on the FFA content of the yolk.

### 2.6. LD Lipolysis generates FFAs in the blastodisc to maintain the desired embryonic ATP levels

**Figure 4.**
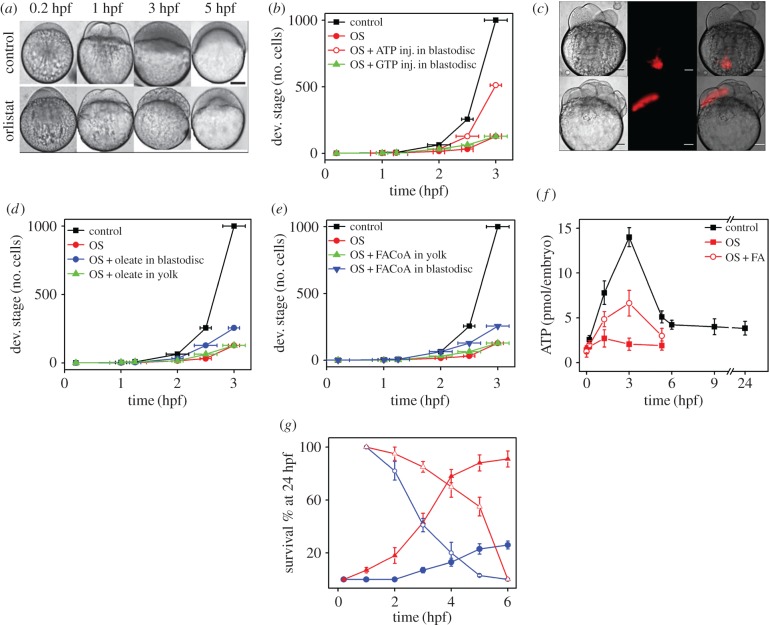
LD metabolism regulates intraembryonic ATP levels crucial for blastomeric protein degradation during early embryonic development. (*a*) Images showing development of control embryos at 0.2, 1, 3 and 5 hpf. The lower panel shows OS-treated embryos at 0.2, 1, 3 and 5 hpf. Note the delayed phenotype in the drug-treated embryos. Scale bar, 200 µm. (*b*) Plot of developmental stage versus time (hpf) to compare the progress of development of control (black), OS-treated (red, closed symbol), OS-treated + ATP injected in the blastodisc (red, open symbol), and OS-treated + GTP-injected (green) embryos. Developmental stage is represented in terms of number of cells at that particular stage. (*c*) DIC, fluorescence and merged images (left to right) for embryos injected with Bodipy-C_12_ into the yolk (upper panel) and blastodisc (lower panel) regions. (*d*) Plot of developmental stage versus time (hpf) to compare the progress of development of control (black), OS-treated (red), OS-treated + Na-oleate injection into the blastodisc (blue) and OS-treated + Na-oleate injection into the yolk (green) embryos. Developmental stage is represented in terms of number of cells at that particular stage. (*e*) Plot of developmental stage versus time (hpf) to compare the progress of development of control (black), OS-treated (red), OS-treated + FACoA injection into the blastodisc (blue) and OS-treated + FACoA injection into the yolk (green) embryos. Developmental stage is represented in terms of number of cells at that particular stage. (*f*) Comparison of total ATP levels (in picomoles/embryo) at same developmental stages (hpf) for control (black), OS-treated (red, closed symbol) and OS-treated Na-oleate injected (red, open symbol) embryos. As OS-treated embryos develop slowly, the developmental stages for OS-treated embryos are plotted corresponding to the respective developmental stages of the control embryos in hpf. (*g*) Plot of survival percentage of embryos at 24 hpf when injected with (closed symbols) for (*t*–∞) or treated with cycloheximide (blue) and OS (red) and then washed off (open symbols) (0–*t*) at different developmental stages of the embryos. All experiments have been done in triplicates and the mean values are plotted with error bars denoting s.e.m. For (*b*,*d*,*e*), the error bar reflects the s.e.m for the duration required to reach specific developmental stage.

Next we explored the link between the rate of development of the zebrafish embryos and the metabolism of LDs. Figure 4*a* compares the developmental stages of control embryos at different time points with those of OS-treated embryos. Inhibition of embryonic lipolysis slows down the rate of development compared with control embryos (figure 4*a,b*). We observe that at 3 hpf, the control embryo is at the 1000-cell stage, while the OS-treated embryo is still at the 256-cell stage (figure 4*a,b*). We also observe that the OS-treated embryos fail to survive beyond 5 hpf. We hypothesize that the inhibition of lipolysis could reduce the level of embryonic ATP, leading to the slower development of OS-treated embryos. To validate this, we performed a rescue experiment on the OS-treated embryos. While injection of ATP into the blastodisc of the embryos rescues the rate of development, the injection of ATP into the yolk of the embryos does not (figure 4*b*; electronic supplementary material, figure S5). The GTP injection has no significant effect on the rate of development in OS-treated embryos (figure 4*b*). Interestingly, injection of ATP in control embryos has no such effect on their rate of development (electronic supplementary material, figure S4). Taken together, the rescue experiments establish the link between the generation of embryonic ATP and lipolysis during early development.

We next hypothesize that the FFAs generated via lipolysis within the blastodisc could act as fuel for the synthesis of the embryonic ATP [14]. To test this hypothesis, we performed the rescue experiment with FFA. Similar to the rescue of embryonic development with ATP, injection of both FFA (sodium oleate) and fatty acyl coA (dodecanoic acyl CoA, FACoA) into the blastodisc rescues the rate of development for OS-treated embryos (figure 4*c–e*). This strengthens our hypothesis that lipolysis during early embryogenesis of zebrafish generates FFAs which are used as fuel for the generation of the embryonic ATP. As neither yolk-FFAs nor yolk-FACoA migrate to the blastodisc until 5 hpf, the injection of either of them into the yolk is not expected to rescue the rate of development. As expected, injection of FFAs and FACoA into the yolk fails to rescue the rate of development of OS-treated embryos (figure 4*c–e*).

### 2.7. LD lipolysis derived FFAs generate a short pulse of higher embryonic ATP levels during early embryogenesis

Next, we demonstrate the link between LD-lipolysis, FFAs and ATP by directly measuring the ATP levels of zebrafish embryos treated with OS with/without exogenous FFA injection. In control embryos, we observe a short pulse (1.25–4 hpf) of higher embryonic ATP levels. The OS treatment abrogates this short pulse of ATP completely; in addition, it reduces the embryonic ATP levels at other developmental stages as well. Interestingly, injection of FFAs into the blastodisc of OS-treated embryos rescues the ATP pulse partially (figure 4*f*).

Next, we investigated the temporal window of the embryonic development in which the lipolysis is vital. For this, we treated the embryos with the drug (OS) for two different but complementary durations of development in a complementary-duration-treatment assay (CDTA). Briefly, the embryos were divided into two sets at a given developmental stage. For example at ‘*t*’ hpf, while the first set was treated with OS from 0 to ‘*t*’ hpf (called 0–*t* treatment), the other set was treated for the complementary duration of embryonic life (i.e. ‘*t*’ hpf onwards; called *t*–∞ treatment; see ‘Experimental procedures’). The survival of these embryos was scored at 24 hpf. Figure 4*g* describes the result of CDTA for OS and cycloheximide (translation inhibitor). We used cycloheximide treatment as a control experiment because protein translation is expected to be vital throughout the entire duration of embryonic development. For cycloheximide, the complementary duration (*t*–∞ treatment), as expected, demonstrates that protein translation is critical at all the developmental stages because just a small fraction of embryos survive even at later *t* (figure 4*g*). While the other set (0–*t* treatment) demonstrates a gradual loss of survival. We notice that cycloheximide treatment of minimum 4 h is required for its action (figure 4*g*). CDTA results of OS suggests that lipolysis is vital during first 3–4 h of embryonic development.

### 2.8. The short embryonic ATP pulse is responsible for active degradation of proteins during OET in zebrafish embryos

**Figure 5.**
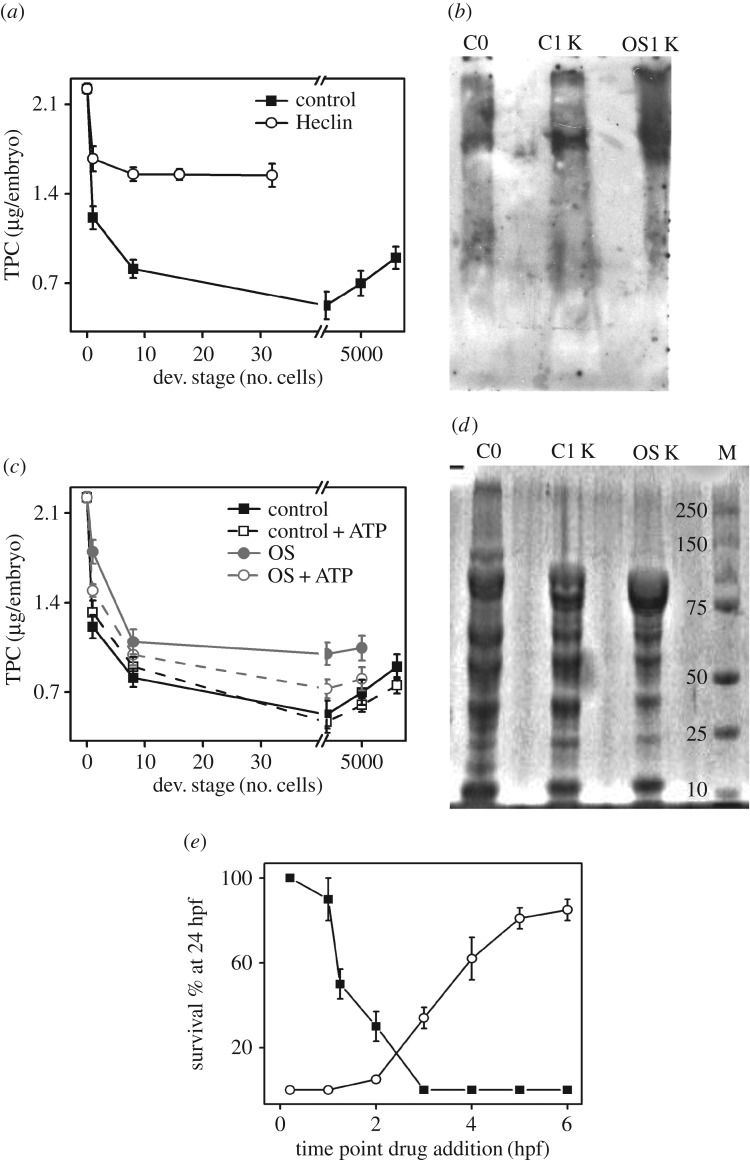
LD metabolism regulates protein degradation during early embryonic development. (*a*) Plot of TPC (µg per embryo) versus developmental stages of control (closed symbol) and Heclin-treated (open symbol) embryos. Note embryos die within 2 hpf post-Heclin treatment. Fifty per cent epiboly is represented on the *x*-axis by 5000 cells. (*b*) Immunoblot using ubiquitin (Ub) antibody for proteins from control embryos at 1-cell stage (C0), 1000-cell stage (C1 K) and OS-treated embryos at 1000-cell stage (OS1 K). Note the difference in the pattern of the Ub-bound proteins in the three conditions (C0, C1 K and OS1 K). (*c*) Plot of TPC (µg/embryo) versus developmental stages for control (black), and Orlistat (grey)-treated embryos. TPC of ATP-injected control (black, dashed line) and ATP-injected OS treated (grey, dashed line) embryos are also plotted against the developmental stages. The developmental stages are represented as the number of cells at that particular developmental stage. Fifty per cent epiboly is represented on the *x*-axis by 5000 cells, while 90% epiboly is represented by 9000 cells in the *x*-axis. (*d*) Protein profile of SDS–PAGE gel stained with CBB G-250. Note the difference in protein pattern among the 1-cell (C0), 1000-cell (C1 K), 1000-cell OS-treated (OS1 K) embryos. The profile of molecular marker is shown in the lane marked M. (*e*) Plot of survival percentage of embryos at 24 hpf when injected with (closed symbols) or treated with (open symbol) Heclin at different developmental stages of the embryos. All experiments have been done in triplicates and the mean values are plotted with error bars denoting s.e.m.

Total protein content (TPC) of the embryos was estimated using the standard Bradford's protein quantification assay [37]. TPC of the control embryos was determined across different developmental stages. As depicted in figure 5*a*, we observe rapid protein degradation by the 8-cell stage (1.25 hpf). TPC of the embryos continues to decrease up to the sphere stage (approx. 4 hpf), beyond which there is a gradual increase in the TPC level (figure 5*a*). This shows that protein degradation accompanies early stages of embryonic development in zebrafish. Degradation of specific proteins like katanin in *C. elegans* embryos during OET is mediated via the ubiquitin proteasomal pathway (UPP) [7]. Therefore, next we investigated whether the OET-associated protein degradation in zebrafish embryos occurs via the ATP-dependent UPP pathway. For this, we compared the TPC of control embryos with that of Heclin (HECT E3 Ub ligase inhibitor)-treated embryos [38]. As the Heclin-treated embryos fail to grow beyond the 32-cell stage (electronic supplementary material, figure S6) and also show morphological defects, we compared TPC until the 32-cell stage (figure 5*a*). Heclin treatment shows an overall increase in the TPC of the embryos at similar developmental stages (figure 5*a*), suggesting that protein degradation during OET in zebrafish embryos is driven by a ATP-dependent UPP pathway. We further confirmed this by comparing the fraction of ubiquitinated embryonic proteins at the 1000-cell stage compared with that of the 1-cell-stage embryo. For this, we loaded same amount of total proteins in the gel. Figure 5*b* shows the western blot depicting the increased fraction of ubiquitinated embryonic proteins at the 1000-cell stage compared with the 1-cell stage. This further confirms that degradation of proteins during early embryogenesis in zebrafish involves UPP pathway.

Next, to explore the link between active protein degradation during OET and pool b ATP, we studied the TPC in OS-treated embryos. Inhibition of lipolysis in zebrafish embryos prevents active protein degradation associated with OET (figure 5*c*). If the gain of TPC in OS-treated embryos is because of the lack of pool b ATP, then injection of ATP into the blastodisc of these embryos must restore the active protein degradation. As hypothesized, injection of ATP indeed restores the protein degradation in the OS-treated embryos, whereas injection of ATP into control embryos has no effect on the protein degradation (figure 5*c*). This suggests that the active protein degradation during OET is pool-b-ATP-dependent and it requires the ATP pulse generated via lipolysis-derived FFAs. As OS treatment alters the protein degradation, we also observe that it alters the level of ubiquitinated embryonic proteins (figure 5*b*) and changes the pattern of Coomassie protein staining (figure 5*d*).

We next carried out the CDTA experiment using Heclin to determine the relevance of active protein degradation accompanying different developmental stages of zebrafish embryos. Figure 5*e* describes the result of CDTA using Heclin. The *t*–∞ treatment using Heclin shows that protein degradation is vital at early developmental stages (up to 3 hpf), however; beyond this, the treatment shows a gradual gain of survival (figure 5*e*). Heclin treatment for both 0–*t* and *t*–∞ sets of embryos points towards the vitality of protein degradation accompanying OET in zebrafish embryos (figure 5*e*).

## 3. Discussion

Lipids are one of the major biochemical components of the cells. Compared with proteins and nucleic acids, the biochemical roles of lipids in cell physiology and embryonic development are poorly explored. For example, cells/embryos contain a large number of lipid forms. What are the *in vivo* functions of these individual lipid forms? How does lipid metabolism contribute to the embryonic development and cell physiology? Many such questions still remain unknown. As the lecithotrophic embryos of zebrafish do not consume any food until 4 dpf, their development is dependent on the maternally supplied nutrients (NLs) in the form of lipids [9]. The maternally supplied lipids must be catabolized into biomolecules that can generate metabolic energy to power the embryonic development. As LDs are the organelles within the blastodisc that can catabolize lipids, we hypothesized that LD-mediated lipolysis must be critical for the early development of zebrafish embryos.

If the embryos use LDs to power early embryogenesis, then the size of the LDs must diminish due to lipolysis. We do observe that lipolysis of LD during first 3 h of development leads to steady decrease in its size. Interestingly, inhibition of lipolysis in the embryos gives rise to LDs larger than the normal size (figure 1*g*). This indicates that there is a continuous growth of the LDs in the zebrafish embryos; however, the activity of cytosolic lipases continuously regulates their size to the set value. The existence of a smaller-sized population of LDs in figure 1*f,g* suggests the existence of other classes of lipases which are not inhibited by OS. We believe that the lipases which are not inhibited by OS overwork to compensate the inhibition of lipolysis, leading to the smaller than normal LDs. This observation also indicates the possibility that different LDs may contain different sets of lipases. The lipolysis of the LDs by the different lipases also leads to alterations in LD-lipid composition during early embryonic development. As reported in our previous work, LDs exhibit two distinct states ‘active’ and ‘inactive’ during the early development of zebrafish embryos [34]. During inactive state, LDs are bigger, circular and their shape is stable, whereas during the ‘active’ state, they are comparatively smaller, irregularly shaped and exhibit enhanced shape fluctuations. Using OS treatment of the embryos, we demonstrate that the periodic shape fluctuation of the LDs is driven by the activity of cytosolic lipases.

**Figure 6.**
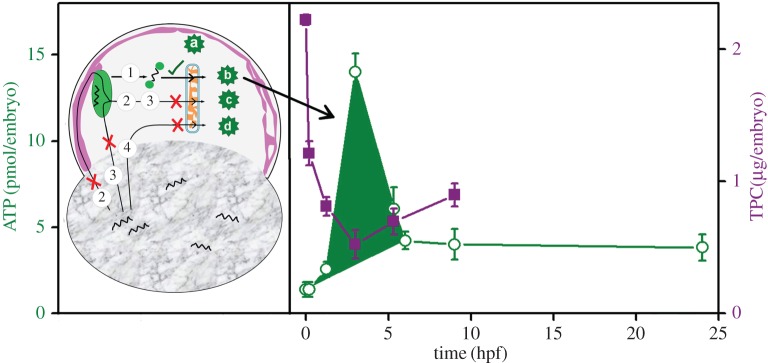
Schematic depiction of embryonic ATP pulse and associated active protein degradation. Left panel shows figure 1*a* again with the source of FFAs (route 1; green tick) that is used for the synthesis of ATP. Red crosses indicate the other possible routes (routes 2, 3, 4) and sources of FFAs that are not used for the synthesis of embryonic ATP until 5 hpf. The right panel depicts the short embryonic ATP pulse (shaded area in green, open symbol, left axis, indicated by the arrow) required to accomplish the ATP-dependent protein degradation (purple solid symbol, right axis) during early embryogenesis.

Literature reports have established that both the trafficking of Bodipy-C_12_ and its incorporation into NLs and PLs is identical to that of radio-labelled C_12_ [39]. Therefore, tagging of FFAs with Bodipy-C_12_ does not alter its *in vivo* trafficking. Thus, if the yolk-FFA migrates to the cytoplasm of the blastodisc via route 2 (figure 1*a*), it would label the core of LDs. However, if the yolk-FFA migrates via route 3, its hydrophobic interaction with LD would label the surface of the LDs. By injection of Bodipy-C_12_ in the yolk and subsequent time-lapse images, we establish that the yolk-FFAs are not exported to the blastodisc until 5 hpf even though we observe a steady increase in FFA in the blastodisc (figure 3*b*). Thus, the FFA requirement within the blastodisc during early zebrafish embryogenesis must be fulfilled by LD-lipolysis (i.e. route 1, figure 1*a*). Using OS treatment, we establish that new pool of FFAs in the blastodisc is generated via route 1 (i.e. the lipolysis of the LDs; figures 1*a* and 6).

As inhibition of lipolysis slows down the rate of embryonic development, we also investigated if the newly synthesized FFA in the blastodisc participates in the synthesis of embryonic ATP. We find that the inhibition of lipolysis reduces the embryonic ATP levels, leading to the slower development of the embryos. Our finding that the injection of ATP rescues the rate of development for OS-treated embryos indicates a possible link between the generation of embryonic ATP and lipolysis during early development. Therefore, we hypothesize that the FFAs generated via lipolysis within the blastodisc could act as fuel for the synthesis of the embryonic ATP. A rescue experiment with injection of FFA and FACoA in the blastodisc further strengthens our hypothesis. On the other hand, failure of the rescue experiment when the FFA or FACoA is injected in yolk strengthens our hypothesis that neither the yolk-FFAs nor the FACoA are exported to the blastodisc during first 5 h of the development. However, we cannot rule out the possibility of existence of other modified forms of FFA which are transported to the blastodisc for the synthesis of ATP.

We also observe the existence of an intraembryonic ATP pulse during the course of embryonic development (figures 4*f* and 6). This in turn depends on the lipolysis of LDs, clearly demonstrating that FFAs derived from LD metabolism are used by the embryos to generate ATP. Thus, the embryos possess two distinct pools of ATP: (i) maternally stored (figure 6, pool a) and (ii) generated by the oxidation of embryonic FFAs (figure 6, pool b, pool c and pool d). As routes 2, 3 and 4 (figure 6) do not work for transport of yolk-FFA, the embryo contains just two pools of ATP, maternally supplied pool a and pool b (figure 6).

Protein degradation during OET is one of the earliest ATP-dependent events that accompany the development as seen in *Caenorhabditis elegans* and *Drosophila* embryos [7]. In zebrafish embryos, the OET involves ATP-dependent degradation of various proteins via the UPP pathway. The maternal pool of ATP (pool a; present in the oocytes) is insufficient to execute UPP. Therefore, a short pulse of additional embryonic ATP (pool b, figure 6) is generated to feed the UPP. Interestingly, only the FFAs generated by the LDs via lipolysis are used for synthesizing the short pulse of additional embryonic ATP.

In conclusion, we demonstrate that the synthesis of LDs and its subsequent lipolysis is very vital for early (until 4 hpf) development of the zebrafish embryos. The early development requires an additional ATP pulse (0–4 hpf) to execute active protein degradation associated with OET. This additional pulse of embryonic ATP is generated in the blastodisc by the β-oxidation of FFAs. The embryonic yolk contains sufficient amount of maternally supplied FFAs yet they are not exported to the blastodisc until 5 hpf. Instead, the LDs in the blastodisc undergo extensive lipolysis to generate FFAs to feed the β-oxidation. Our studies also indicate that maternally supplied pool of ATP is insufficient to power even the early development. This highlights the importance of LDs in development of zebrafish embryos.

## 4. Experimental procedures

### 4.1. Embryo culture, handling and drug treatments

Wild-type *Danio rerio* was purchased from a local market and all experiments were carried according to the guidelines of the Indian Association for the Cultivation of Science, Animal Ethics Committee. The fish were maintained at 28°C and an alternate 12 L : 12 D cycle was maintained under a water circulation system (Aquatic Habitats Inc.). The fish were bred in a 2 : 1 male : female ratio. Embryos were collected in embryo media, E3 (50 mM Nacl, 0.17 mM KCl, 0.33 mM CaCl_2_, 0.33 mM MgSO_4_). OS (Orlistat, O-STAT 120, Aristo) was used at a concentration of 150 µM in E3 medium. Both Heclin (Tocris Bioscience) and Cycloheximide (Sigma) were used at a concentration of 7 µM in E3 medium.

### 4.2. Injection of Na-oleate, dodecanoic acetyl CoA ammonium salt, bodipy-C_12_ into the embryos

Injection volume for all samples was maintained between 3 and 5 nl. Na-oleate (Tocris Biosciences) was used at a concentration of 1 mM. Dodecanoic acetyl CoA ammonium salt (Avanti Polar Lipids) was also used at a concentration of 1 mM. Ten micrometre solution of BODIPY-C_12_ (BODIPY 558/568 C_12_; 4,4-difluoro-5-(2-thienyl)-4-bora-3a,4a-diaza-*s*-indacene-3-dodecanoic acid; Molecular Probes) was prepared and injected into either the yolk or the blastodisc as mentioned in figure 3*c*.

### 4.3. LD isolation and lipid extraction

A total of 150 embryos at each developmental stage were collected and dechorionated using Pronase followed by deyolking [40]. The blastodisc fraction was homogenized in 250 mM sucrose in T-buffer by syringe plunging and pestle-based homogenization. The lysate was then ultra-centrifuged at 10 000*g* for 1 h at 4°C in a sucrose gradient of 70, 55, 50 and 15% in T-buffer [31]. To determine LD fraction, each of the layers was stained with NR (electronic supplementary material, figure S1). Lipid extraction from the LDs was carried out using standard chloroform: methanol-based procedure [41].

### 4.4. Lipid type identification

Lipids extracted from LDs were run on TLC Silica gel 60 plates (Merck) using both neutral (for apolar lipids) and polar (for phospholipids) solvents. To char the TLC plates, they were sprayed with a solution of 10% copper (II) sulfate in 10% phosphoric acid solution and heated with a hot air gun. NLs resolve better in neutral solvent of *n*-hexane/diethyl ether/acetic acid (60 : 40 : 1, v/v/v), while the polar lipids were resolved using polar solvent chloroform/methanol/ammonia solution 25% (65 : 25 : 4, v/v/v). The *R*_f_ values for each spot (i.e. the length of the migration of a particular spot to the migration of the solvent front) are determined. Referring to table 2, we identify the lipid class of each spot based on its *R*_f_ value.

### 4.5. FFA estimation

The blastodisc and yolk were collected in separate tubes from 30 embryos. Both samples were homogenized in T-buffer followed by lipid extraction. The extracted lipid samples were run in duplicates in TLC plates. One set was charred to identify the location of FFAs; the other set was used to isolate FFAs from the same corresponding location in set 1. A 1 cm TLC plate strip encompassing FA spot (*R*_f_ *∼* 0.56) was scraped out from set 2. The FFA was recovered by extraction with *n*-hexane and diethyl ether. Dried FFA powder was weighed to quantify the FFA content.

### 4.6. Protein and ATP-level estimation

Embryos were dechorionated, deyolked and homogenized in protein lysis buffer (10 mM Tris–Cl pH 8, 150 mM NaCl, 0.1% SDS, 1% NP-40, 0.5% protease inhibitor (Sigma)) on ice. The protein content of the lysate was assayed using standard Bradford assay. The absorbance of the samples was recorded at 595 nm wavelength using a spectrophotometer (Molecular Devices Spectromax M2). Absorbance was then converted to TPC µg/embryo with the help of a standard curve using bovine serum albumin (BSA) at different concentrations as the protein samples. ATP content of the embryos was assessed using Promega ENLITENATP kit. For this, the embryo lysates were mixed with phenol-TE/chloroform/de-ionized water followed by repeated centrifugation to release the ATP into the supernatant. The ATP content was read at 560 nm using a luminometer (Thermo Scientific Luminoskan Ascent).

### 4.7. Image acquisition and image processing and developmental stage identification of the embryos

Time-lapse imaging of LDs at every 3 s interval was carried out with Olympus BX61 upright microscope. The images were segmented using ImageJ 1.47t. Area and perimeter of the LDs was determined for each frame. The ‘circularity’ parameter which defines shape of the LDs was then evaluated using the formula
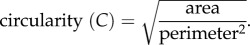


where *C* ranges from 0 to 1 with 1 depicting maximum circularity. The circularity values were determined for LDs across successive frames and time traces of normalized circularity, 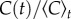 was calculated.

For imaging the fate of BODIPY-C_12_ injected into yolk of the embryos, alternate DIC and fluorescence images (542 nm excitation) were acquired at every 10 min interval up to 3 hpf using an inverted microscope (Zeiss Axio Observer Z1).

For SEM imaging, isolated LDs were fixed using high-grade Glutaraldehyde (Sigma) for 2 h. They were treated with 4% OsO_4_ followed by dehydration using increasing concentration of alcohol followed by SEM imaging (Jeol JSM-6700F). The size of the LDs was measured using a DLS instrument, Zetasizer NanoZS (Malvern Particle Size Analyzer).

Time-lapse images of the embryos starting at 0.2 hpf (1-cell stage) until 3 hpf (1024-cell stages in control) were obtained to study the rate of embryonic development. The developmental stages (in number of cells) of the embryos were assigned by comparing the above-acquired image with the reference images from the Zebrafish Information Network (ZFIN). It can also be compared with Kimmel *et al.*'s work [42]. The average time required to reach a given developmental stage is estimated from these time-lapse images.

### 4.8. Complementary duration treatment assay

Freshly laid embryos were collected at 1-cell stage and these were divided into two separate batches. One set was incubated with the drug of interest (OS/Heclin/Cycloheximide) from initial time point (*t* = 0) and the drug was washed off from these embryos at different time points post-treatment (*t* hpf) and the survival percentage of the embryos was calculated at 24 hpf (0–*t* treatment). The second set of embryos was treated with the drug of interest from specific developmental stage (*t* hpf) and kept for incubation up to 24 hpf when their survival percentage was assayed (*t*–∞).

### 4.9. SDS–PAGE-based protein separation

Fifty embryos per sample were homogenized using protein lysis buffer and 2 µg of each sample loaded in a 10% sodium dodecyl sulfate–polyacrylamide gel electrophoresis (SDS–PAGE) gel. A molecular marker (Biorad Precision Plus) was also loaded along with the samples. The gel was treated with staining solution containing Coomassie Brilliant Blue G-250 (CBB G-250) and then destained and imaged using Gel-Doc XR (Bio-Rad).

### 4.10. Immunoblot analysis

Two micrograms of each protein were loaded and separated by a 10% SDS–PAGE gel. This was electro-transferred to a PVDF membrane. The membrane was incubated with anti-Ubiquitin antibody (Cell Signaling Technology). This was then treated with peroxidase-conjugated anti-rabbit IgG polyclonal antibody (Cell Signaling Technology). The membrane was washed and incubated with chemiluminescent substrate followed by exposure to the photographic film and visualized post development.

## Supplementary Material

Supplementary information

## Data accessibility

The datasets supporting this article have been uploaded as part of the electronic supplementary material.
