# Supplementary material for: Zebrafish lipid droplets regulate embryonic ATP homeostasis to power early development
Source: Open Biol. 2017 Jul 5;7(7):170063. doi: 10.1098/rsob.170063 (PMC5541346; doi:10.1098/rsob.170063)
Supplement: Supplementary information [file rsob170063supp1.docx]

**Zebrafish lipid droplets regulate embryonic ATP homeostasis to power early development**

Asmita Dutta, Deepak Kumar Sinha^[[1]](#footnote-1)^

Indian Association for the Cultivation of Science

Department of Biological Chemistry

Jadavpur, Kolkata-700032, India

DOI: 10.1098/rsob.2016xxxx

**Supplementary Information**

**
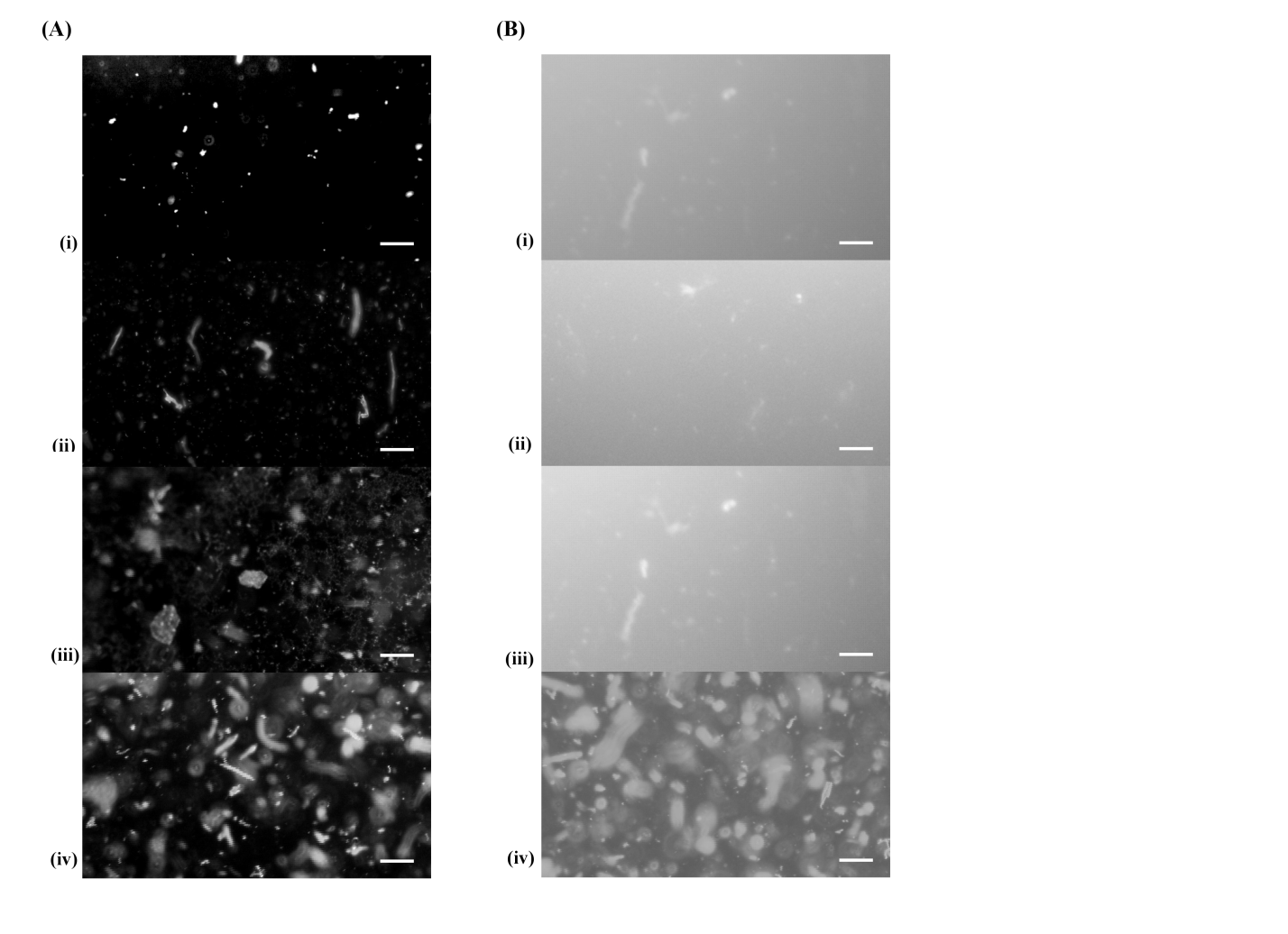
**

**Figure S1. Fluorescence images of Nile Red stained sucrose gradient layers obtained post ultra-centrifugation of blastodisc and yolk fractions.**

To determine the appropriate layer which will contain the LDs post sucrose gradient based LD isolation; we separated the blastodisc and the yolk regions of 150 embryos and carried out LD isolation from both the regions separately. Post this, each layer was viewed using Olympus BX61 upright microscope with 10 X objective and under green excitation light.

(A) Blastodisc layers post centrifugation viewed under microscope. We find that (i) uppermost layer contains LDs abundantly. (ii) Second layer also contains LDs but debris is also present. (iii) & (iv) are mostly membrane debris. (B) Yolk layers post centrifugation viewed under microscope. We find that none of the layers (i-iv) contains any distinct LD-like structures even though they show a very high fluorescence signal depicting the presence of high amounts of lipids in them. Scale bar 3 µm.

**
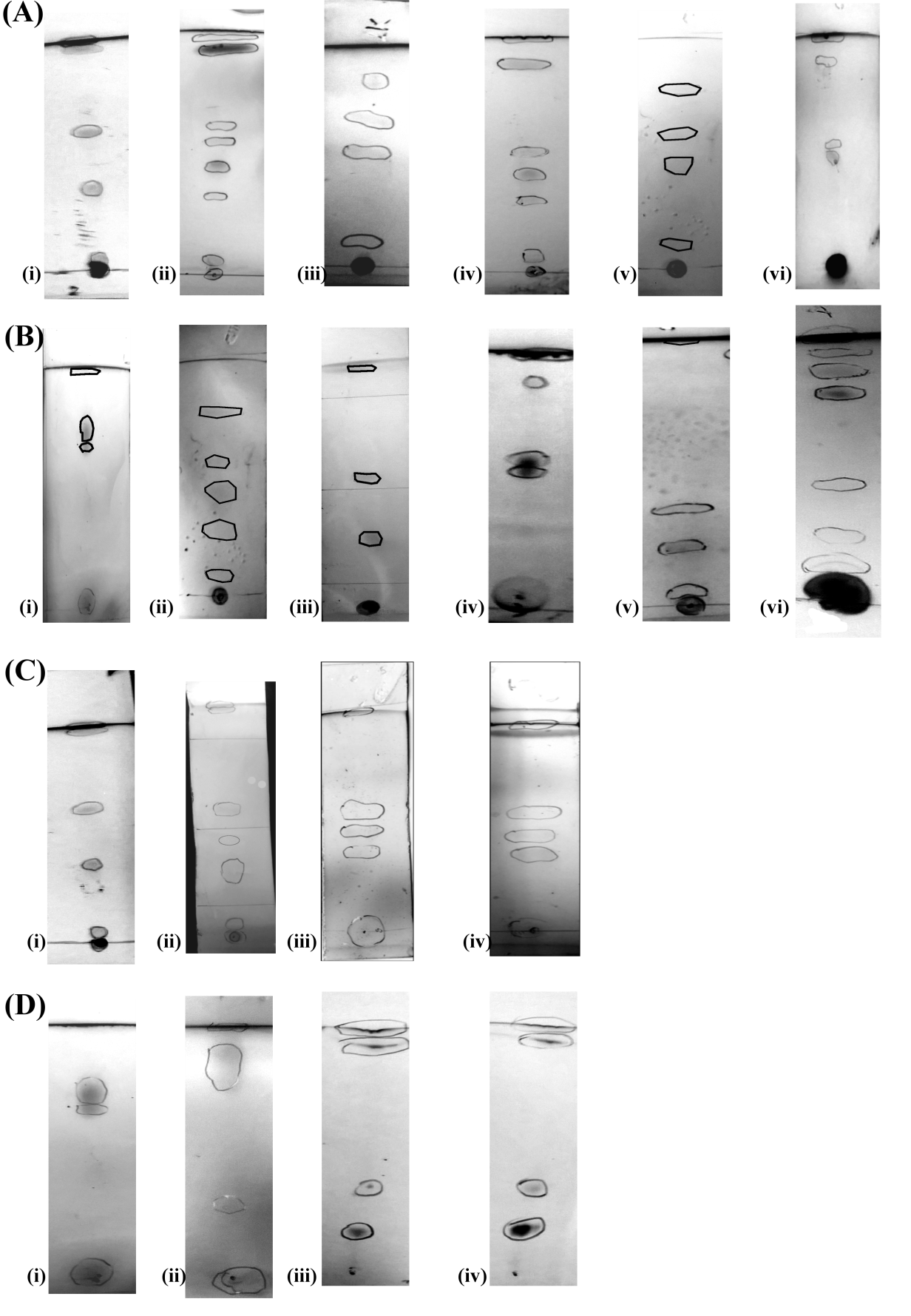
**

**Figure S2. Lipolysis drives the alterations in the lipid profile of LDls with embryonic development.**

(A) TLC profiles of lipids extracted from LDs that were isolated from control embryos at (i) 0.2 hpf, (ii) 1.25 hpf, (iii) 3hpf, (iv) 5.3 hpf, (v) 9 hpf and (vi) 24 hpf developmental stages and run in a neutral solvent system.

(B) TLC profiles of lipids extracted from LDs that were isolated from control embryos at (i) 0.2 hpf, (ii) 1.25 hpf, (iii) 3hpf, (iv) 5.3 hpf, (v) 9 hpf and (vi) 24 hpf developmental stages and run in a polar solvent system.

(C) TLC profiles of lipids extracted from LDs that were isolated from OS treated embryos at (i) 0.2 hpf, (ii) 1.25 hpf, (iii) 3hpf and (iv) 5.3 hpf developmental stages and run in a neutral solvent system.

(D) TLC profiles of lipids extracted from LDs that were isolated from OS treated embryos at (i) 0.2 hpf, (ii) 1.25 hpf, (iii) 3hpf and (iv) 5.3 hpf developmental stages and run in a polar solvent system.


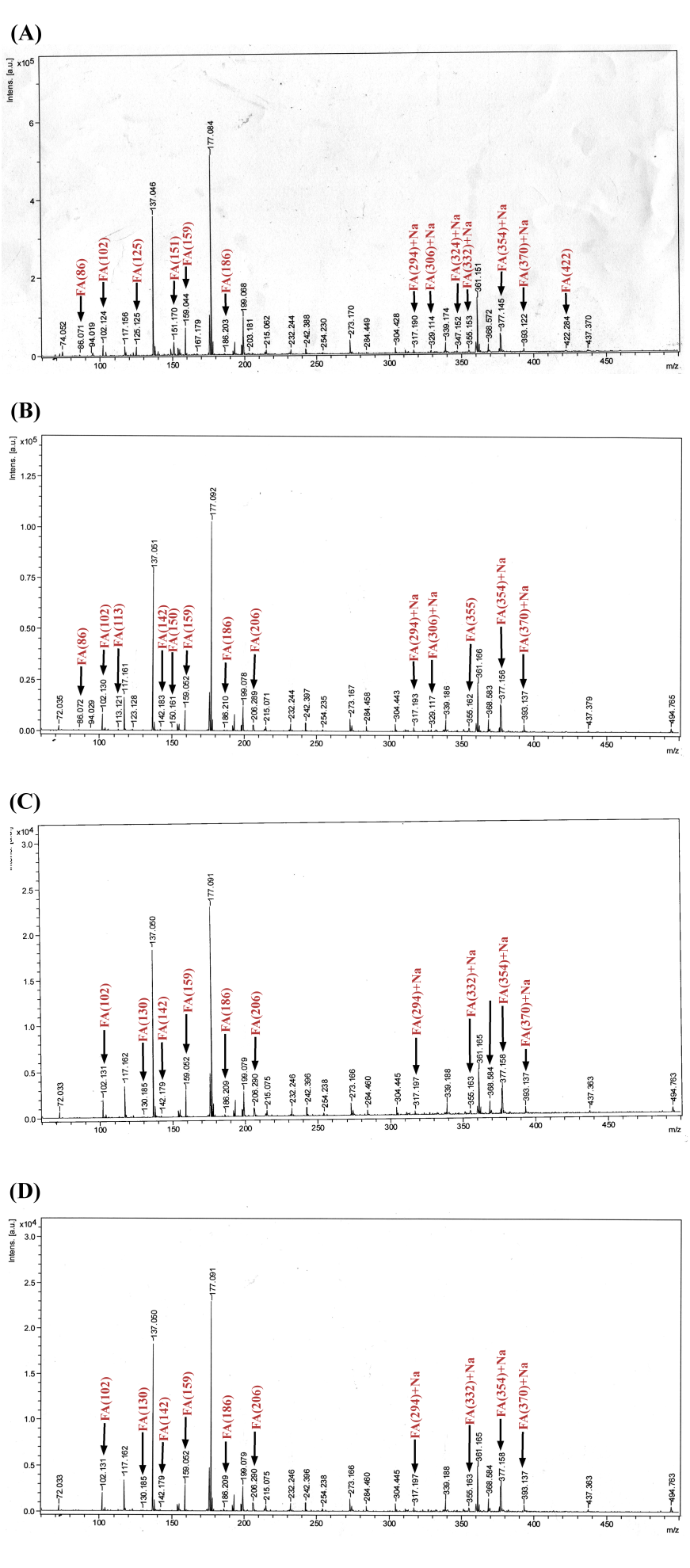


**Figure S3.** **FA spot scraped out from TLC plates depending upon their R*_f_* values contain principally FAs.**

MALDI-TOF MS data sheets for FA spots obtained from control and OS treated embryos. (A) MALDI data sheet for FAs from the blastodisc of control embryos, (B) MALDI data sheet for FAs from the yolk of control embryos, (C) MALDI data sheet for FAs from the blastodisc of OS treated embryos and (D) MALDI data sheet for FAs from the yolk of OS treated embryos. The arrows point to the peaks corresponding to the FAs as determined by the LIPIDOMICS GATEWAY database.

**
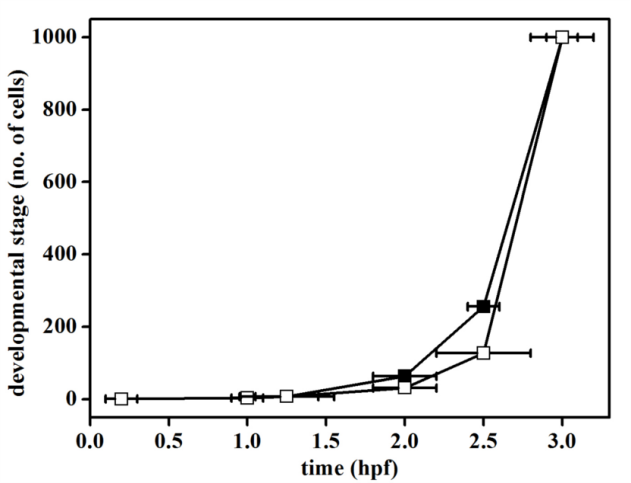
**

**Figure S4. Injection of exogenous ATP into control embryos does not alter embryonic development.**

Plot of development stage versus the time (hpf) for control embryos (closed symbol) and ATP injected control embryos (open symbol). Experiment has been done in triplicates and the mean has been plotted with the S.E.M.


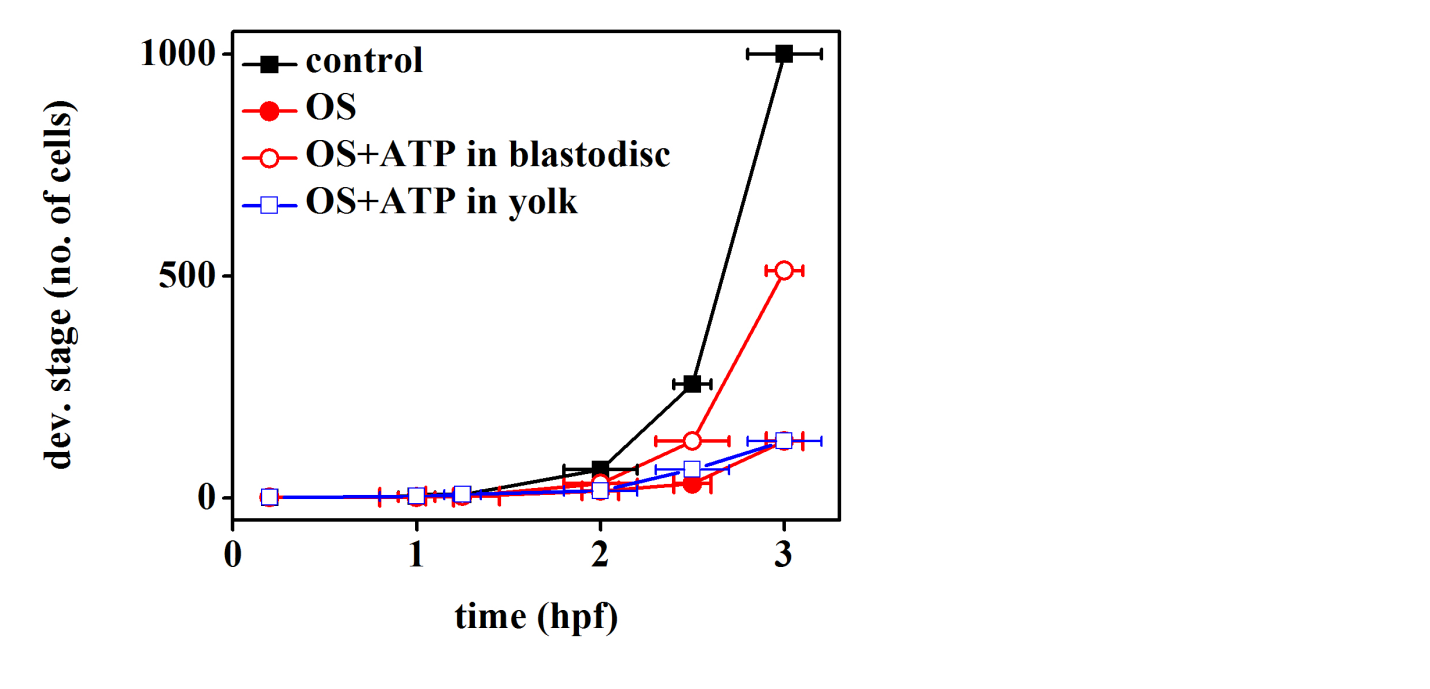


**Figure S5.** Plot of developmental stage versus time (hpf) to compare the progress of development of control (black), OS treated (red, closed symbol), OS treated + ATP injected in the blastodisc (red, open symbol), and OS treated + ATP injected in the yolk (blue) embryos. Developmental stage is represented in terms of no. of cells at that particular stage.

**
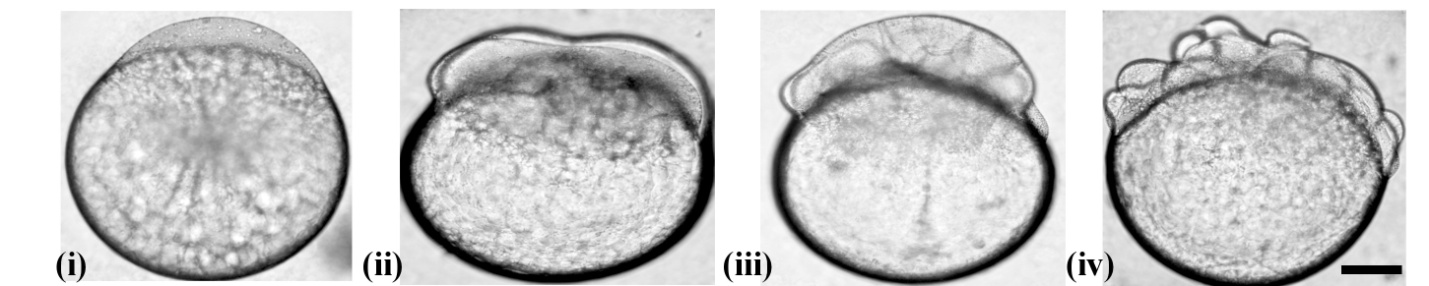
**

**Figure S6. Heclin treatment slows down the embryonic development and causes morphological deformations of the embryos.**

Embryos treated with Heclin were imaged at (i) 0.2 hpf, (i) 1.25 hpf, (iii) 1.5 hpf, (iv) 2 hpf. The Heclin treated embryos did not survive post 2 hpf. Scale bar is 200 µm.

**Table S1**

| **FA from Control Blastodisc** | | **FA from Control Yolk** | | **FA from OS Blastodisc** | | **FA from OS Yolk** | |
| --- | --- | --- | --- | --- | --- | --- | --- |
| **m/z** | **Subclass of FAs** | **m/z** | **Subclass of FAs** | **m/z** | **Subclass of FAs** | **m/z** | **Subclass of FAs** |
| 86 | Unsaturated FA and conjugates FA0102 (4 members) | 86 | Unsaturated FA and conjugates FA0102 (4 members) | 102 | Straight chain/ branched FAs FA0101, FA0102 ( 10 members) | 102 | Straight chain/ branched FAs FA0101, FA0102 ( 10 members) |
| 102 | \| Straight chain/ branched FAs FA0101, FA0102 ( 10 members) \|  \|  \|  \|  \|  \|  \| \| --- \| --- \| --- \| --- \| --- \| --- \| --- \| | 102 | \| Straight chain/ branched FAs FA0101, FA0102 ( 10 members) \|  \| \| --- \| --- \| | 130 | Straight/ branched chain FAs FA0101, FA0102 (14 members) | 130 | Straight/ branched chain FAs FA0101, FA0102 (14 members) |
| 125 | Unsaturated FA and conjugates FA01 (2 members) | 113 | -- | 142 | Branched/ unsaturated FAs FA0102, FA0103 (28 members) | 142 | Branched/ unsaturated FAs  FA0102, FA0103 (28 members) |
| 151 | Branched/ Unsaturated FA and conjugates FA0102, FA0103 (12 members) | 142 | Branched/ unsaturated FAs FA0102, FA0103 (28 members) | 159 | Unsaturated FA FA0103 (1member) | 159 | Unsaturated FA FA0103 (1member) |
| 159 | Unsaturated FA FA0103 (1member) | 150 | Branched/ Unsaturated FA and conjugates FA0102, FA0103 (12 members) | 186 | Straight chained/branched/unsaturated FAs and conjugates FA0101, FA0102, FA0103, FA0105 (20 members) | 186 | Straight chained/branched/unsaturated FAs and conjugates FA0101, FA0102, FA0103, FA0105 (20 members) |
| 186 | Straight chained/branched/unsaturated FAs and conjugates FA0101, FA0102, FA0103, FA0105 (20 members) | 159 | Unsaturated FA FA0103 (1member) | 206 | Hydroxy FAs FA0105 (2 members) | 206 | Hydroxy FAs FA0105 (2 members) |
| 203 | Straight chain FA FA0101 (1 member) | 206 | Hydroxy FAs FA0105 (2 members) | 317 | Branched/unsaturated FAs FA0102, FA0103 (6 members) | 317 | Branched/unsaturated FAs FA0102, FA0103 (6 members) |
| 317 | Branched/unsaturated FAs FA0102, FA0103 (6 members) | 317 | Branched/unsaturated FAs FA0102, FA0103 (6 members) | 355 | Unsaturated FAs FA0103 (3 members) | 355 | Unsaturated FAs FA0103 (3 members) |
| 329 | Branched/unsaturated FAs  FA0102, FA0103 (17 members) | 329 | Branched/unsaturated FAs  FA0102, FA0103 (17 members) | 368 | ---- | 368 | ---- |
| 347 | Branched/ unsaturated FAs FA0102, FA0103 (7 members) | 355 | Unsaturated FAs FA0103 (3 members) | 377 | Straight chain/  branched chain/ unsaturated FAs FA0101, FA0102, FA0103 (4 members) | 393 | Hydroxy FA FA0105 (1 member) |
| 355 | Unsaturated FAs FA0103 (3 members) | 377 | Straight chain/branched chain/unsaturated FAs FA0101, FA0102, FA0103 (4 members) |  |  |  |  |
| 377 | Straight chain/branched chain/unsaturated FAs FA0101, FA0102, FA0103 (4 members) | 393 | Hydroxy FA FA0105 (1 member) |  |  |  |  |
| 393 | Hydroxy FA FA0105 (1 member) | 494 | ---- |  |  |  |  |
| 422 | Branched/ unsaturated FAs FA0102, FA0103 (6 members) |  |  |  |  |  |  |

**Table S1.** **FA spot from TLC plate corresponds to FA as verified by MALDI-TOF.** Details of the identity of the FA peaks obtained from the MALDI-TOF MS data sets. The m/z peaks obtained were searched in the LIPIDOMICS GATEWAY database and the classes of the lipids have been mentioned in the table above.

1. bcdks@iacs.res.in [↑](#footnote-ref-1)
